# Supplementary material for: How high-intensity sensory consumption fills up resource scarcity: The boundary condition of self-acceptance
Source: PLoS One. 2023 May 26;18(5):e0285853. doi: 10.1371/journal.pone.0285853 (PMC10218729; doi:10.1371/journal.pone.0285853)
Supplement: S1 File — (ZIP) [file pone.0285853.s001.zip › Supporting information(Compressed ZIP)/S4 Appendix D.docx]

**Appendix D**

High self-Acceptance condition

1. I’m not a bad person when I act badly; I am a person who has acted badly.

2. I’m not a good person when I act well and accomplish things; I am a person who has acted well and accomplished things.

3. I can accept myself whether I win, lose, or draw.

4. I would better not define myself entirely by my behavior, by others’ opinions, or by anything else under the sun.

5. I can be myself without trying to prove myself.

6. I have many faults and can work on correcting them without blaming, condemning, or damning myself for having them.

7. I can neither prove myself to be a good nor a bad person. The wisest thing I can do is simply to accept myself.

8. I can itemize my weaknesses, disadvantages, and failures without judging or defining myself by them.

9. Seeking self-esteem or self-worth leads to self-judgments and eventually to self-blame. Self-acceptance avoids these self-ratings.

10. I can reprimand my behavior without reprimanding myself.

11. I can praise my behavior without praising myself.

12. Get after your behavior! Don’t get after yourself.

13. I can acknowledge my mistakes and hold myself accountable for making them—but without berating myself for creating them.

14. It’s silly to favorably judge myself by how well I’m able to impress others, gain their approval,

perform, or achieve.

15. It’s equally silly to unfavorably judge myself by how well I’m able to impress others, gain their approval, perform, or achieve.

16. I do not have to let my acceptance of myself be at the mercy of my circumstances.

17. I am not the plaything of others’ reviews and can accept myself apart from others’ evaluations of me.

18. I may at times need to depend on others to do practical things for me, but I don’t have to emotionally depend on anyone in order to accept myself. Practical dependence is a fact! Emotional dependence is a fiction!

19. I am beholden to nothing or no one in order to accept myself.

20. It may be better to succeed, but success does not make me a better person.

21. It may be worse to fail, but failure does not make me a worse person.

Low self-Acceptance condition

List of Daily Activities

1. Eating breakfast.

2. Watching TV.

3. Doing homework.

4. Listening to music.

5. Using the computer.

6. Going to the park.

7. Going to the movies.

8. Going shopping.

9. Going for a pleasure drive.

10. Riding a bike.

11. Eating lunch.

12. Studying for class.

13. Taking a trip or vacation.

14. Calling a friend.

15. Snacking.

16. Engaging in my hobby.

17. Going to the gym.

18. Eating dinner.

19. Visiting a family member.

20. Starting my workday.

21. Going to bed.
