# Supplementary material for: How high-intensity sensory consumption fills up resource scarcity: The boundary condition of self-acceptance
Source: PLoS One. 2023 May 26;18(5):e0285853. doi: 10.1371/journal.pone.0285853 (PMC10218729; doi:10.1371/journal.pone.0285853)
Supplement: S1 File — (ZIP) [file pone.0285853.s001.zip › Supporting information(Compressed ZIP)/S5 Appendix E.docx]

**Appendix E**

1.It’s unbearable to fail at important things, and I can’ t stand not succeeding at them.

2.When people who I want to like me disapprove of me or reject me, I can’ t bear their disliking me.

3.It’s unbearable being uncomfortable, tense, or nervous, and I can’ t stand it when I am.

4.It is awful and terrible to be treated unfairly by people in my life.

5.If important people dislike me, it is because I am an unlikable, bad person.

6.If people treat me without respect, it goes to show how bad they really are.

7.I have worth as a person even if I do not perform well at tasks that are important to me.
