# Supplementary material for: How high-intensity sensory consumption fills up resource scarcity: The boundary condition of self-acceptance
Source: PLoS One. 2023 May 26;18(5):e0285853. doi: 10.1371/journal.pone.0285853 (PMC10218729; doi:10.1371/journal.pone.0285853)
Supplement: S1 File — (ZIP) [file pone.0285853.s001.zip › Supporting information(Compressed ZIP)/S6 Appendix F.docx]

**Appendix F**

1. When walking through stores, I can’t help touching all kinds of products.

2. Touching products can be fun.

3. I place more trust in products that can be touched before purchase.

4. I feel more comfortable purchasing a product after physically examining it.

5. When browsing in stores, it is important for me to handle all kinds of products.

6. If I can’t touch a product in the store, I am reluctant to purchase the product.

7. I like to touch products even if I have no intention of buying them.

8. I feel more confident making a purchase after touching a product.

9. When browsing in stores, I like to touch lots of products.

10. The only way to make sure a product is worth buying is to actually touch it.

11. There are many products that I would only buy if I could handle them before purchase.

12. I find myself touching all kinds of products in stores.
