# Supplementary figures and images for: How high-intensity sensory consumption fills up resource scarcity: The boundary condition of self-acceptance
Source: PLoS One. 2023 May 26;18(5):e0285853. doi: 10.1371/journal.pone.0285853 (PMC10218729; doi:10.1371/journal.pone.0285853)

Appendix A The auditory equipment


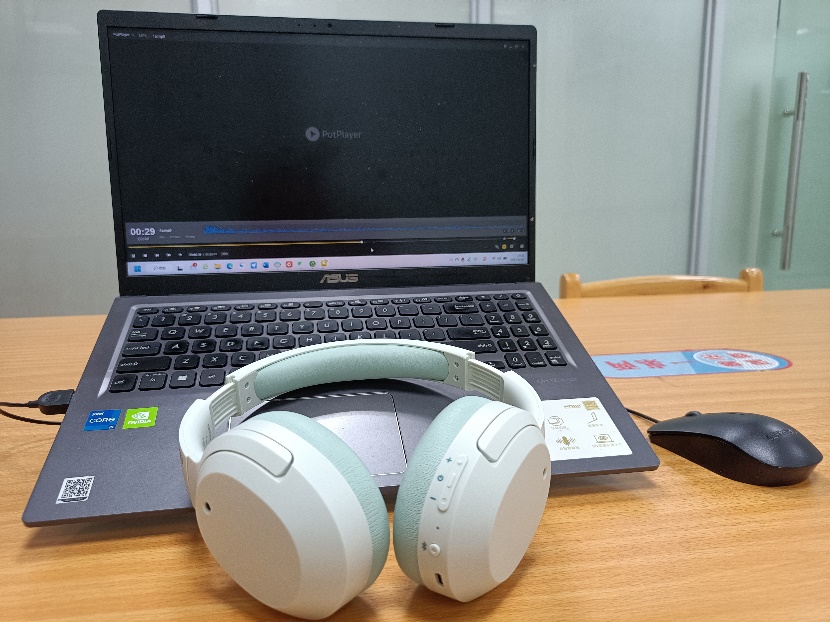

Supplement: S1 File — (ZIP) [file pone.0285853.s001.zip › Supporting information(Compressed ZIP)/S1 Appendix A.docx]

Appendix B The decibel meter


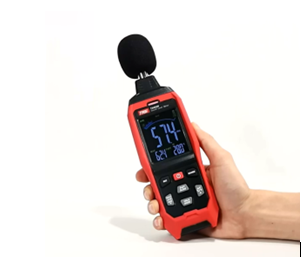

Supplement: S1 File — (ZIP) [file pone.0285853.s001.zip › Supporting information(Compressed ZIP)/S2 Appendix B.docx]

Appendix C The professional instrument of colorimeters


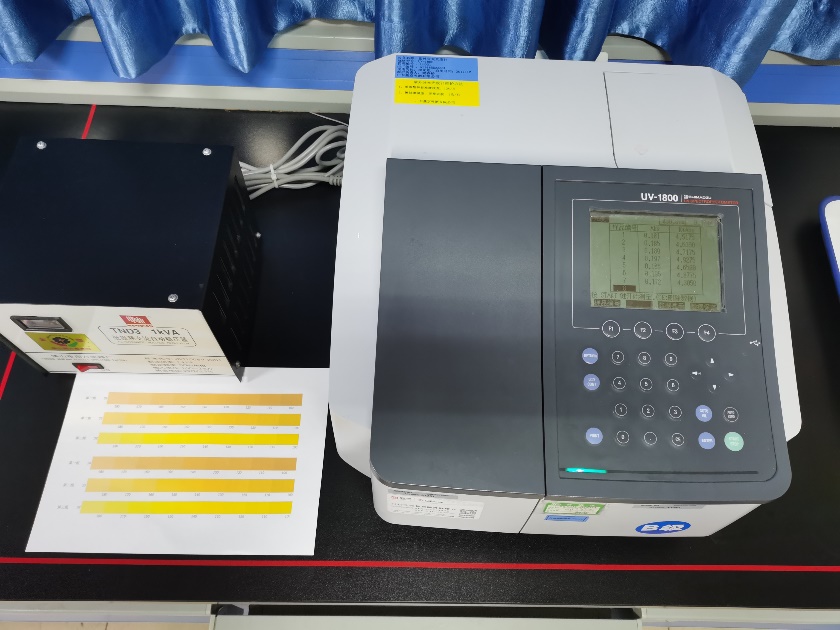

Supplement: S1 File — (ZIP) [file pone.0285853.s001.zip › Supporting information(Compressed ZIP)/S3 Appendix C.docx]
