# Supplementary material for: How high-intensity sensory consumption fills up resource scarcity: The boundary condition of self-acceptance
Source: PLoS One. 2023 May 26;18(5):e0285853. doi: 10.1371/journal.pone.0285853 (PMC10218729; doi:10.1371/journal.pone.0285853)
Supplement: Attachment — Submitted filename: renamed_e972f.docx [file pone.0285853.s002.docx]

**Manuscript ID:** PONE-D-22-28511

**Manuscript title:** How high-intensity sensory consumption fills up resource scarcity: The boundary condition of self-acceptance

**To: PLOS ONE Editor**

**Re: Response to reviewers**

Dear Editor and reviewers,

Thank you for allowing a resubmission of our manuscript, with an opportunity to address the reviewer comments.

We have uploaded on the system (a) our point-by-point responses to comments (responses to the reviewer and editor), (b) the revised manuscript with the changes shown, and (c) the cleanly updated manuscript with no changes shown if the submission system is accessible.

In this revised manuscript, we added a coauthor Haiyan Luo to participate in the review of the manuscript to improve the quality of the manuscript.

Best regards

Liangjun Peng, Yuxin Peng, Haiyan Luo, Yeying Den

**Response to Reviewer 1**

**Reviewer#1, Concern # 1:** **The design of experiment (DOE) was quite interesting having said that the auditory equipment and color intensity instrument were lacking.**

**Author Response:** In Section 3.1 Methodology, we explain that all participants used the same model and make of computer and headphones to listen to the music clips without lyrics. So, the auditory equipment used in our experiments were computers and headphones. In the revised manuscript, we added auditory equipment. The color swatches of high- and low- intensity visual stimuli in the experiment were indeed modulated by chemical engineers using professional colorimeters. We did not emphasize this process in the first manuscript. Thank the reviewer for finding that we are missing the description of colorimeter. In the revised draft, we added the description of colorimeter which was omitted in the first manuscript.

**Author action:**

(1) In Section 3.1 Methodology, we update the manuscript as follows: Further, for the purposes of this study, brand new headphones were purchased and tested by the assistant across all computers to ensure the same quality of audibility (the auditory equipment see Appendix A).

(2) In Section 5.1 Methodology, we update the manuscript as follows: The color swatches of high- and low- intensity visual stimuli in our experiment were modulated by chemical engineers using professional instrument of colorimeters (the instrument see Appendix B)

**Reviewer#1, Concern # 2: This study would be even better with a proper instrumentation as this could be collaborated with other transdisciplinary personnel (pity it did not happen). I would possibly reject this paper as it is lacking of that simple arrangement. However, other angle is considered quite interesting to taken into consideration as this might be explored even more.**

**Author Response:** As mentioned earlier, we used some necessary equipment and instruments in our experiment, such as colorimeter, decibel meter, computer, earphone, etc., which are not emphasized in the manuscript. We collaborate with other interdisciplinary people to use these devices, like color palette engineers and audiologists. In the revised manuscript, we have added the missing equipment, such as colorimeter, decibel meter, computer, and earphone, and illustrated the equipment drawings in Appendix A, B, and C.

**Author action:** In Section 3.1 Methodology, we update the manuscript as follows: We used a decibel meter to record decibel levels at different volumes, and the record showed that the higher the volume, the higher the decibel level (the decibel meter see Appendix C). Previous studies also show that the increased volume of auditory stimuli corresponds to increased decibel levels and more intensive sound (Gelfand 2009).

**Reviewer#1, Concern # 3:** **The sampling distribution are quite unacceptable and the participant involved could be classified into generic parameters (e.g., educational background/traumatic event/sex/races/ages/lifestyle etc.).**

**Author Response:** We consider the validity of the sample distribution in the experiment and we collect demographic data about the participants in each experiment. In the revised manuscript, the the participants involved have been classified into generic parameters (eg, educational background/sex/ages/major., etc.).

**Author action:**

**(1)** At the section 3.1 Methodology, we updated the manuscript as follows: Ninety-six undergraduate students and teachers from the Business School participated in the experiment, including 49 females (51.04%) and 47 males (48.96%) ranging from 18 to 35-years-old with a mean age of 20 (SD=2.421).

**(2)** At the section 4.1 Methodology, we updated the manuscript as follows: One hundred and ninety-one participants from college students and teachers were randomly assigned to two groups of positive and negative valence conditions. A total of 191 students and teachers participated in the experiment, including 93 females (48.70%) and 98 males (51.30%) ranging from 18 to 46-years-old with a mean age of 21.06 (SD=4.34). 95 (48 females, 50.53%) participated in the negative valence conditions and 96 (47 females, i.e., 48.96%) in the positive valence conditions.

**(3)** In Section 5.1 Methodology, we updated the manuscript as follows: We recruited 182 voluntary participants from an online research service platform(www.credamo.com) called Credamo who were paid RMB 1.5 (USD 25 cents) for the online experiment. The final valid sample comprised 160 participants (82 female i.e., 51.3%, average age 31.34, ranging from 18 to 62 with SD=9.648) who were randomly assigned to the conditions of resource scarcity and control condition, after excluding those incomplete responses. 80 (39 females, 48.75%) participated in resource scarcity conditions and 80 (43 females, that is, 53.75%) in control conditions. Among the 160 participants, 25 (15.63%) had master's degree, 99 (61.88%) had bachelor's degree and 36 (22.50%) had junior college degree or below.

**(4)** In Section 6.1 Methodology, we update the manuscript as follows: Experiment 3 was still conducted on the Credamo platform (www.credamo. com), with a total of 251 voluntary participants who were paid RMB 1.5 (USD 25 cents) for the online experiment. The final valid sample comprised 240 participants (154 female i.e. 64.17%, average age 29.21, ranging from 18 to 59 with SD=7.836) who were randomly assigned to the conditions of a 2 (resource: scarcity vs. abundance) x 2 (self-acceptance: high vs. low) between-subjects design. Among the 240 participants, 38 (15.80%) had master's degree, 154 (64.20%) had bachelor's degree and 48 (20%) had junior college degree or below.

**Reviewer#1, Concern # 4: What is your control sampling?**

**Author Response:** Thank you for your comment on the control sampling in our manuscript. We appreciate the opportunity to clarify this aspect of our study.

(1) We selected the control group from a population of individuals who were matched to the study group based on educational background, age, sex, and other demographic factors.

(2) The control group consisted of individuals who were similar to the experimental group in terms of educational background, age, gender, and other relevant factors. This allows us to determine whether any changes observed in the experimental group are due to antecedent variables or other factors.

(3) Eligibility for the control group was determined based on the absence of any other significant factor conditions that could potentially confound the results.

(4) We recognize the importance of using an appropriate control group to ensure the validity and reliability of our findings, and we are confident that the control sampling method we used is appropriate for the research question being investigated.

(5) We will provide the following additional details and justification for this method in the revised manuscript to further clarify the study design.

**Author action:**

**(1)** In the section 3.1 Methodology, we updated the manuscript as follows: Ninety-six undergraduate students and teachers from the Business School participated in the experiment, including 49 females (51.04%) and 47 males (48.96%) ranging from 18 to 35-years-old with a mean age of 20 (SD=2.421).

**(2)** In the section 4.1 Methodology, we updated the manuscript as follows: One hundred and ninety-one participants from college students and teachers were randomly assigned to two groups of positive and negative valence conditions. A total of 191 students and teachers participated in the experiment, including 93 females (48.70%) and 98 males (51.30%) ranging from 18 to 46-years-old with a mean age of 21.06 (SD=4.34). 95 (48 females, 50.53%) participated in the negative valence conditions and 96 (47 females, i.e., 48.96%) in the positive valence conditions.

**(3)** In Section 5.1 Methodology, we updated the manuscript as follows: We recruited 182 voluntary participants from an online research service platform(www.credamo.com) called Credamo who were paid RMB 1.5 (USD 25 cents) for the online experiment. The final valid sample comprised 160 participants (82 female i.e., 51.3%, average age 31.34, ranging from 18 to 62 with SD=9.648) who were randomly assigned to the conditions of resource scarcity and control condition, after excluding those incomplete responses. 80 (39 females, 48.75%) participated in resource scarcity conditions and 80 (43 females, that is, 53.75%) in control conditions. Among the 160 participants, 25 (15.63%) had master's degree, 99 (61.88%) had bachelor's degree, and 36 (22.50%) had junior college degree or below.

**(4)** In Section 6.1 Methodology, we update the manuscript as follows: Experiment 3 was still conducted on the Credamo platform (www.credamo. com), with a total of 251 voluntary participants who were paid RMB 1.5 (USD 25 cents) for the online experiment. The final valid sample comprised 240 participants (154 female i.e., 64.17%, average age 29.21, ranging from 18 to 59 with SD=7.836) who were randomly assigned to the conditions of a 2 (resource: scarcity vs. abundance) x 2 (self-acceptance: high vs. low) between-subjects design. Among the 240 participants, 38 (15.80%) had master's degree, 154 (64.20%) had bachelor's degree and 48 (20%) had junior college degree or below.

**Response to Reviewer 2**

**Reviewer#2, Concern # 1: The moderated mediation in Study 3 needs more explanation and the results should be described in more details. Currently, there are only explanations regarding the moderation effects. What is important to know here is if the self-worth only mediates the effect for the low-acceptance subsample?**

**Author response:** In the revised manuscript, we explain that is if self-worth only mediates the effect for the low-acceptance subsample.

**Author action:** In Section 6.2 Results, we update the manuscript as follows: Specifically, moderated mediation revealed a significant mediation effect of self-worth for low self-acceptance participants (b=- .68, SE= .16, 95% CI= [-1.02, -.38]), but not for high self-acceptance participants (b=.01, SE= .03, 95% CI= [-.04, .06]). This indicates that the self-worth only mediates the effect for the low self-acceptance participants.

**Reviewer#2, Concern # 2: Generally, it is possible that there is a risk for a certain tautology when using self-worth and self-acceptance as mediator and moderator. The authors should explain the difference in more detail and discuss how it is possible that there is a strong connection but still room for these different processes.**

**Author Response:** Thank you very much for your comment. In the revised version, we explain the difference in more detail and discuss how it is possible that there is a strong connection but still room for these different processes.

**Author action:** In Section 2.4 Self-acceptance, we updated the manuscript as follows: Self-worth and self-acceptance are two related but distinct concepts in psychology that play different roles in regulating our thoughts, emotions, and behaviour [92, Crocker et al., 2004]. Self-worth refers to our overall sense of value and worth as a person, while self-acceptance refers to our ability to acknowledge and accept all parts of ourselves, including our flaws and weaknesses [63, Wei et al., 2011]. Self-worth can be seen as a mediator in the sense that it can influence how we feel about ourselves and how we behave in response to different situations [75]. For example, if someone has high self-worth, they may be more confident in their abilities and less likely to experience negative emotions such as anxiety or self-doubt. Self-acceptance, on the other hand, can be seen as a moderator in the sense that it can influence the relationship between other factors and our sense of self-worth [Neff et al., 2009]. For example, if someone has a high level of self-acceptance, they may be less likely to be affected by negative feedback or criticism from others, which can help to protect their self-worth. On the contrary, if someone has low self-acceptance, they may be more vulnerable to negative feedback and criticism, which can undermine their sense of self-worth. In short, although self-worth and self-acceptance are closely related, these different processes still have independent room for operation, which highlights the importance of understanding these two concepts to influence consumer behaviour differences [Brenner et al., 2017].

**Reviewer#2, Concern # 3: The authors should reflect and discuss about the relation/processes regarding the self-worth restauration and the HISC with negatively valanced stimuli. For the stimulation with positive sensory input the effect is easily understandable, but it is quite difficult to see a reason why people experiencing scarcity leading to fewer self-worth should be interested in increasing their arousal through negative valanced stimuli.**

**Author Response:** This is indeed a little difficult to understand, but in the revised manuscript, we explained why people experiencing scarcity leading to fewer self-worth should be interested in increasing their arousal through negative valanced stimuli based on previous research findings.

**Author action:** In Section 2.2 Negatively valenced HISC, we updated the manuscript as follows: Prior literature suggests that people experiencing different kinds of scarcity may be interested in enhancing their arousal to restore self-worth through negative valanced stimuli. Griskevicius et al. (2013) find that people experiencing economic deprivation were more likely to seek entertainment with negatively valanced stimuli and suggest that this preference may be a way for people to increase their arousal levels and temporarily escape scarce-related stress and anxiety in order to restore their self-worth. Mogilner et al. (2018) find that people experiencing time scarcity are more inclined to choose products or experiences that offer instant gratification and excitement, even if those experiences are negative in nature. This preference for negatively valanced stimuli, they explain, may be driven by a desire for arousal in the face of limited resources. Meier et al.(2012) find that people who experience food scarcity prefer to view vague stimuli as threats and show higher physiological responses to negatively valanced stimuli. They suggest that this increased sensitivity to negatively valanced stimuli may be a way people respond to potential threats. These studies suggest that people experiencing scarcity may be more interested in negatively valenced stimuli to increase their arousal levels and cope with the stress and anxiety associated with scarcity to restore their self-worth.
