# Supplementary material for: How high-intensity sensory consumption fills up resource scarcity: The boundary condition of self-acceptance
Source: PLoS One. 2023 May 26;18(5):e0285853. doi: 10.1371/journal.pone.0285853 (PMC10218729; doi:10.1371/journal.pone.0285853)
Supplement: Attachment — Submitted filename: renamed_bc74c.docx [file pone.0285853.s003.docx]

**Manuscript ID:** PONE-D-22-28511

**Manuscript title:** How high-intensity sensory consumption fills up resource scarcity: The boundary condition of self-acceptance

**To: PLOS ONE Editor**

**Re: Response to reviewers**

Dear Editor and reviewers,

Thank you for allowing a resubmission of our manuscript, with an opportunity to address the reviewer comments.

We have uploaded on the system (a) our point-by-point responses to comments (responses to the reviewer and editor), (b) the revised manuscript with the changes shown, and (c) the cleanly updated manuscript with no changes shown if the submission system is accessible.

Thanks again to the reviewers for their professional and constructive comments, which are very helpful to improve our research.

Best regards

Liangjun Peng, Yuxin Peng, Haiyan Luo, Yeying Den

**Response to Reviewer 1**

**Reviewer#1, Concern # 1:** **Abstract: No. of participant were not disclosed and the DOE was quite simplified and more towards a vague arrangement.**

**Author Response:** First, we fully disclosed the participants in the Abstract section in revised manuscript. Second, we elaborate the DOE of Abstract section (including participants, variable measurement methods, statistical analysis methods, experimental results) so that it is clearly presented to the reader. Third, we reinforced the DOE in the first paragraph of each study.

**Author action:**

**(1) In the methods section of the Abstract, we update the manuscript as follows:**

We used different methods including one-way ANOVA, linear regression, mediating effect, and moderating effect to test the four hypotheses. Four experiments in the study were conducted from May 2022 and August 2022 and involved undergraduates from a university and volunteers recruited online. All participants are adults and verbally agree to participate voluntarily. Study 1a (**N = 96** (male 47, female 49), participants from a business school in China) measured resource scarcity in the laboratory experiments and verified the effect of resource scarcity on consumer HISC preference by using linear regression (H1). Study 1b (**N = 191** (male 98, female 93), students and teachers from a university in China) measured resource scarcity in the laboratory experiments and manipulated positively and negatively valenced experiences. Using the PROCESS SPSS Mode l, we verified that negatively valenced stimuli also lead to higher levels of arousal, which in turn restores the self-discrepancy caused by resource scarcity (H2). Study 2 (an online experiment, **N = 182** (male 91, female 91), participants from China) manipulated resource scarcity in color sensory stimulant context, replicating the preliminary effect and examined the mediating effect of the self-worth by using the PROCESS SPSS Mode 4 (H3). Study 3 (an online experiment, **N = 251** (male 125, female 126), participants from China) manipulated resource scarcity and self-acceptance in tactile sensory experience, and tested the moderating effect of self-acceptance by using the PROCESS SPSS Mode 8 (H4).

**(2) We reinforced the DOE in the first paragraph of each study as follows:**

**Study 1a** was designed to test H1 by using linear regression method to study the effect of resource scarcity on consumers’ audio consumption choices. Study 1a was conducted in a laboratory and recruited 96 participants from a business school in China. We measured participants' perceived resource scarcity in laboratory experiments.

An important goal that **study 1b** was designed to test H2 by using PROCESS SPSS Mode l method to examine what effect the valence of HISC may have on the observed impacts. Study 1a was conducted in a laboratory and recruited 191 students and teachers from a business school in China. We manipulated participants' positively and negatively valenced experiences.

The design of **Study 2** is to test H3 by using PROCESS SPSS Mode l method to document the mediating role of self-worth between resource scarcity and HISC in the visual domain. Study 2 was conducted on a research service online platform (www.credamo.com) called Credamo and recruited 182 voluntary participants. We manipulated participants' resource scarcity in color sensory stimulant context.

The design purpose of **study 3** is to test H4 by using PROCESS SPSS Mode 8 to investigate the moderating effect of self-acceptance on resource-scarce individuals’ preference for HISC in the tactile domain. Study 3 was conducted on a research service online platform (www.credamo.com) called Credamo and recruited 251 voluntary participants. We manipulated participants' resource scarcity and self-acceptance in tactile sensory experience.

**Reviewer#1, Concern # 2: Page 12: The no. of participant was only mentioned at this section (182 participant) and this should be clearly worth mentioning earlier for a better understanding.**

**Author Response:** We fully understand the reviewer's comments. As in Reply to Concern # 1, we advance participants to the first paragraph of each study (Study 1a, Study 1b, Study 2, Study 3) because this should be clearly worth mentioning earlier for a better understanding.

**Author action:** we advance participants to the first paragraph of each study (Study 1a, Study 1b, Study 2, Study 3) as follows:

Study 1a was designed to test H1 by using linear regression method to study the effect of resource scarcity on consumers’ audio consumption choices. Study 1a was conducted in a laboratory and recruited **96 participants** from a business school in China. We measured participants' perceived resource scarcity in laboratory experiments.

An important goal that study 1b was designed to test H2 by using PROCESS SPSS Mode l method to examine what effect the valence of HISC may have on the observed impacts. Study 1a was conducted in a laboratory and recruited **191 students and teachers** from a business school in China. We manipulated participants' positively and negatively valenced experiences.

The design of Study 2 is to test H3 by using PROCESS SPSS Mode l method to document the mediating role of self-worth between resource scarcity and HISC in the visual domain. Study 2 was conducted on a research service online platform (www.credamo.com) called Credamo and recruited **182 voluntary participants**. We manipulated participants' resource scarcity in color sensory stimulant context.

The design purpose of study 3 is to test H4 by using PROCESS SPSS Mode 8 to investigate the moderating effect of self-acceptance on resource-scarce individuals’ preference for HISC in the tactile domain. Study 3 was conducted on a research service online platform (www.credamo.com) called Credamo and recruited **251 voluntary participants**. We manipulated participants' resource scarcity and self-acceptance in tactile sensory experience.

**Reviewer#1, Concern # 3: The specific statistical analysis in the method section was quite unclear and need more info on that.**

**Author Response:** We have revised the method part of the abstract, in which we separately added the participants, the variable measurement method, and the **specific** statistical analysis method of each experiment. The specific statistical analysis methods used in experiment 1a, experiment 1b, experiment 2 and experiment 3 are linear regression, **PROCESS SPSS Mode l**, **PROCESS SPSS Mode 4,** and **PROCESS SPSS Mode 8,** respectively.

**Author action:** we added the **specific statistical analysis in the method section** as follows:

We used different methods including **one-way ANOVA, linear regression, mediating effect, and moderating effect** to test the four hypotheses. Four experiments in the study were conducted from May 2022 and August 2022 and involved undergraduates from a university and volunteers recruited online. All participants are adults and verbally agree to participate voluntarily. Study 1a (N = 96 (male 47, female 49), participants from a business school in China) measured resource scarcity in the laboratory experiments and verified the effect of resource scarcity on consumer HISC preference by using **linear regression** (H1). Study 1b (N = 191 (male 98, female 93), students and teachers from a university in China) measured resource scarcity in the laboratory experiments and manipulated positively and negatively valenced experiences. Using the **PROCESS SPSS Mode l**, we verified that negatively valenced stimuli also lead to higher levels of arousal, which in turn restores the self-discrepancy caused by resource scarcity (H2). Study 2 (an online experiment, N = 182 (male 91, female 91), participants from China) manipulated resource scarcity in color sensory stimulant context, replicating the preliminary effect and examined the mediating effect of the self-worth by using the **PROCESS SPSS Mode 4** (H3). Study 3 (an online experiment, N = 251 (male 125, female 126), participants from China) manipulated resource scarcity and self-acceptance in tactile sensory experience, and tested the moderating effect of self-acceptance by using the **PROCESS SPSS Mode 8** (H4).

**Reviewer#1, Concern # 4: The ultimatum comment: The DOE need to be clearly explained and visualized (e.g. Table and Figures) so that the reviewer could understanding at the fullest of the outcome of this studies. Not to mention it is quite excruciating lengthy with unclear statistical approaches.**

**Author Response:** **First,** we revised the method section of the Abstract, which clearly explains the design of experiment for each study including participants, variable measurement methods, statistical analysis methods. **Second,** we revised the first paragraph of study 1a, study 1b, study2, and study3, which clearly explains the design of experiment including the purpose of the experiment, participants, variable measurement methods, statistical analysis methods

**Third,** each of our studies has visual research results, which are included in the result section. Outcome of this studies are visualized in 10 Figures, which uploaded separately according to journal requirements and are not included in the manuscript. In order to enable reviewer to understand at the fullest of the outcome of this studies, we show the figures below.

**Author action:**

**First,** we update the method section of the Abstract as follows:

We used different methods including one-way ANOVA, linear regression, mediating effect, and moderating effect to test the four hypotheses. Four experiments in the study were conducted from May 2022 and August 2022 and involved undergraduates from a university and volunteers recruited online. All participants are adults and verbally agree to participate voluntarily. Study 1a (N = 96 (male 47, female 49), participants from a business school in China) measured resource scarcity in the laboratory experiments and verified the effect of resource scarcity on consumer HISC preference by using linear regression (H1). Study 1b (N = 191 (male 98, female 93), students and teachers from a university in China) measured resource scarcity in the laboratory experiments and manipulated positively and negatively valenced experiences. Using the PROCESS SPSS Mode l, we verified that negatively valenced stimuli also lead to higher levels of arousal, which in turn restores the self-discrepancy caused by resource scarcity (H2). Study 2 (an online experiment, N = 182 (male 91, female 91), participants from China) manipulated resource scarcity in color sensory stimulant context, replicating the preliminary effect and examined the mediating effect of the self-worth by using the PROCESS SPSS Mode 4 (H3). Study 3 (an online experiment, N = 251 (male 125, female 126), participants from China) manipulated resource scarcity and self-acceptance in tactile sensory experience, and tested the moderating effect of self-acceptance by using the PROCESS SPSS Mode 8 (H4).

**Second,** we update the first paragraph of study 1a, study 1b, study2, and study3 as follows:

**Study 1a** was designed to test H1 by using linear regression method to study the effect of resource scarcity on consumers’ audio consumption choices. Study 1a was conducted in a laboratory and recruited 96 participants from a business school in China. We measured participants' perceived resource scarcity in laboratory experiments. We examine the propensity for HISC by participants’ attitude toward an intense (high decibel) sound and their preferred volume on continuous audio in the same audio clips ranging from less intense to high intense sound (low decibel to high decibel). The audio sound we used in study 1a is music clips without lyrics.

An important goal that **study 1b** was designed to test H2 by using PROCESS SPSS Mode l method to examine what effect the valence of HISC may have on the observed impacts. Study 1a was conducted in a laboratory and recruited 96 students and teachers from a business school in China. We manipulated participants' positively and negatively valenced experiences. Unlike study 1a, which used only positively valenced sensory stimuli, study 1b included negatively valenced stimuli. We expect that individuals subjected to resource scarcity prefer negatively valenced stimuli just as they prefer positively valenced stimuli, since negatively valenced stimuli also lead to higher levels of arousal, which in turn restores the self-discrepancy caused by resource scarcity.

In studies 1a and 1b, we demonstrate that individuals subjected to resource scarcity exhibit a propensity for HISC in the auditory domains. We speculate that resource scarcity would reduce an individual’s self-worth and that HISC itself, like other affirming activities, serves as a restorative mechanism of self-worth [105] (Steele 1988; Townsend, Sood 2012). The design of **Study 2** is to test H3 by using PROCESS SPSS Mode l method to document the mediating role of self-worth between resource scarcity and HISC in the visual domain. Study 2 was conducted on a research service online platform (www.credamo.com) called Credamo and recruited 182 voluntary participants. We manipulated participants' resource scarcity in color sensory stimulant context. We hypothesize that resource scarcity reduces the individual’s self-worth, which in turn promotes an individual’s HISC preference and ultimately has a restorative effect on self-worth. Unlike studies 1a and 1b, in study 2 we examine the propensity for HISC by the attitude of the participants toward intense color (highly saturated) and by their preferred choice of color on a continuum of shades ranging from less intense (less saturated) to more intense colors (more saturated) in the same hue family.

The design purpose of **study 3** is to test H4 by using PROCESS SPSS Mode 8 to investigate the moderating effect of self-acceptance on resource-scarce individuals’ preference for HISC in the tactile domain. Study 3 was conducted on a research service online platform (www.credamo.com) called Credamo and recruited 251 voluntary participants. We manipulated participants' resource scarcity and self-acceptance in tactile sensory experience. Study 3 shows that individuals subjected to resource scarcity prefer HISC in the visual domain when they are not allowed additional opportunities to restore their self-worth, which is mediated by self-worth. First, Study 3 is designed to investigate the robustness of the effects observed in previous studies by testing whether resource scarcity impacts the preference for HISC in the tactile domain. Second, Study 3 will expand on previous studies by testing whether self-acceptance attenuates the mediating effect of Study 3.

**Third,** Outcome of this studies are visualized in 10 Figures, which are shown below.

**Fig. 1.** Conceptual framework.

**Fig. 2** Moderating effect of valence (negative vs. positive) on the resource scarcity and HISC

**Fig. 3.** Orange color swatches of high- and low- intensity visual stimuli

**Fig. 4** Spectrum of low-high intensity color

**Fig. 5.** The mediating effect of sense of the self-worth.

**Fig. 6** Moderating effect of self-acceptance on the resource scarcity and self-worth

**Fig. 7** Moderating effect of self-acceptance on the resource scarcity and HISC
